# Delirium prediction in the ICU: designing a screening tool for preventive interventions

**DOI:** 10.1093/jamiaopen/ooac048

**Published:** 2022-06-10

**Authors:** Anirban Bhattacharyya, Seyedmostafa Sheikhalishahi, Heather Torbic, Wesley Yeung, Tiffany Wang, Jennifer Birst, Abhijit Duggal, Leo Anthony Celi, Venet Osmani

**Affiliations:** Critical Care Services, Mayo Clinic, Jacksonville, Florida, USA; Fondazione Bruno Kessler Research Institute, Trento, Italy; University of Trento, Trento, Italy; Department of Pharmacy, Cleveland Clinic, Cleveland, Ohio, USA; National University of Singapore, Singapore, Singapore; Division of Pulmonary, Critical Care and Sleep Medicine, Beth Israel Deaconess Medical Center, Boston, Massachusetts, USA; Physical and Occupational Therapy, Mayo Clinic, Jacksonville, Florida, USA; Respiratory Institute, Cleveland Clinic, Cleveland, Ohio, USA; Division of Pulmonary, Critical Care and Sleep Medicine, Beth Israel Deaconess Medical Center, Boston, Massachusetts, USA; Institute of Medical Engineering and Science, Massachusetts Institute of Technology, Boston, Massachusetts, USA; Department of Biostatistics, Harvard T.H. Chan School of Public Health, Boston, Massachusetts, USA; Fondazione Bruno Kessler Research Institute, Trento, Italy

**Keywords:** delirium, clinical decision support, machine learning, artificial intelligence, nursing assessment, predictive modeling

## Abstract

**Introduction:**

Delirium occurrence is common and preventive strategies are resource intensive. Screening tools can prioritize patients at risk. Using machine learning, we can capture time and treatment effects that pose a challenge to delirium prediction. We aim to develop a delirium prediction model that can be used as a screening tool.

**Methods:**

From the eICU Collaborative Research Database (eICU-CRD) and the Medical Information Mart for Intensive Care version III (MIMIC-III) database, patients with one or more Confusion Assessment Method-Intensive Care Unit (CAM-ICU) values and intensive care unit (ICU) length of stay greater than 24 h were included in our study. We validated our model using 21 quantitative clinical parameters and assessed performance across a range of observation and prediction windows, using different thresholds and applied interpretation techniques. We evaluate our models based on stratified repeated cross-validation using 3 algorithms, namely Logistic Regression, Random Forest, and Bidirectional Long Short-Term Memory (BiLSTM). BiLSTM represents an evolution from recurrent neural network-based Long Short-Term Memory, and with a backward input, preserves information from both past and future. Model performance is measured using Area Under Receiver Operating Characteristic, Area Under Precision Recall Curve, Recall, Precision (Positive Predictive Value), and Negative Predictive Value metrics.

**Results:**

We evaluated our results on 16 546 patients (47% female) and 6294 patients (44% female) from eICU-CRD and MIMIC-III databases, respectively. Performance was best in BiLSTM models where, precision and recall changed from 37.52% (95% confidence interval [CI], 36.00%–39.05%) to 17.45 (95% CI, 15.83%–19.08%) and 86.1% (95% CI, 82.49%–89.71%) to 75.58% (95% CI, 68.33%–82.83%), respectively as prediction window increased from 12 to 96 h. After optimizing for higher recall, precision and recall changed from 26.96% (95% CI, 24.99%–28.94%) to 11.34% (95% CI, 10.71%–11.98%) and 93.73% (95% CI, 93.1%–94.37%) to 92.57% (95% CI, 88.19%–96.95%), respectively. Comparable results were obtained in the MIMIC-III cohort.

**Conclusions:**

Our model performed comparably to contemporary models using fewer variables. Using techniques like sliding windows, modification of threshold to augment recall and feature ranking for interpretability, we addressed shortcomings of current models.

## INTRODUCTION

The diagnosis of delirium is common in critically ill patients and depending on the patient population its incidence can be up to 80%.^1^ Typically delirium rates have ranged between 10% and 23%, and half of them acquire delirium in the intensive care unit (ICU).^2^ Delirium leads to increased hospital length of stay and need for prolonged institutionalization for critically ill patients.^3–5^ Delirium drives up healthcare costs, and its impact often persists beyond the ICU including risk for functional decline in daily living activities, and long-term cognitive impairment.^6–10^

Treatment and prevention of delirium is dependent on identifying the complex interplay of multiple triggers in the ICU.^11^ A multimodal strategy of evidence-based best-practice recommendations aimed at coordinating multidisciplinary care to reduce delirium risk and expedite ICU discharge commonly referred to as the ABCDEF bundle is effective in both preventing and treating delirium.^12^^,^^13^ This bundle outlines in detail how we assess, prevent and manage pain, perform both spontaneous awakening and breathing trials daily in intubated and mechanically ventilated patients, choose analgesic and sedative agents, assess, prevent and manage delirium, incorporate early mobility, and engage family members in the care of these critically ill patients. Unfortunately, this bundle of interventions requires education of caregivers, coordination between a multidisciplinary team, is labor and resource intensive, and therefore not consistently implemented across all ICU patients and all health care settings^12^^,^^14^ A screening tool to prioritize ABCDEF implementation to those who are most vulnerable can be an invaluable tool to maximize the benefit of the resource-intensive preventive measures.

Current assessment tools, such as the Confusion Assessment Method for the Intensive Care Unit (CAM-ICU), only diagnoses delirium after its onset.^15^ Administering CAM-ICU requires specialized training. Although each hospital has its own protocol for delirium, because of its time-consuming nature CAM-ICU is infrequently done compared to other vital signs and diagnosis can be delayed. Although certain patient characteristics, such as age, illness severity, and certain medications, are considered high risk for development of delirium or while elevations in inflammatory biomarkers possibly associated with severe disease, these risk factors have been inconsistent in their ability to predict the onset of delirium.^16–18^

Previous prediction models trained on small patient cohorts lacked adequate power to capture the complex relationships between delirium and the time-varying predictor variables.^19^^,^^20^ In attempting to improve the performance, larger administrative datasets were used to develop prediction models, using several hundred variables. However, these models lack interpretability, and are almost impossible to adopt in day-to-day practice.^21^ Additionally, most of these models are not specific to the critically ill population and cannot be extrapolated to the ICU.^20^

We propose to build a screening tool for delirium by developing and fine tuning a delirium prediction model that requires fewer variables than existing models, using large development and validation cohorts in comparison to the existing literature^18^ and that can predict the risk of delirium in a continuous fashion using a sliding window. Using both conventional machine learning methods and deep learning algorithms, we will evaluate performance of our model across various observation and prediction windows to address the issues of variability across time and treatment effects. In addition, we will rank the independent variables in order of their predictive importance to help with interpretability. These attributes should help pave the way for implementation of a screening tool to help caregivers at the bedside.

## METHODS

### Ethical review

The analysis using the eICU Collaborative Research Database (eICU-CRD) is exempt from institutional review board approval due to the retrospective design, lack of direct patient intervention, and the security schema, for which the re-identification risk was certified as meeting safe harbor standards by an independent privacy expert (Privacert, Cambridge, MA, USA) (Health Insurance Portability and Accountability Act Certification no. 1031219-2). The data in the Medical Information Mart for Intensive Care version III (MIMIC-III) are de-identified, and the institutional review boards of the Massachusetts Institute of Technology (No. 0403000206) and Beth Israel Deaconess Medical Center (2001-P-001699/14) both approved the use of the database for research.

### Study population

The eICU-CRD is a freely available multicenter database comprising 200 859 patient unit encounters for 139 367 unique patients admitted between 2014 and 2015 in over 200 hospitals located throughout the United States.^22^ The MIMIC-III database is an open-access single-center ICU database including 53 423 distinct hospital admissions for 46 476 unique patients admitted from 2001 to 2012.^23^ Both datasets comprise data on patient demographics, vitals, clinical flowsheets, laboratory values, medications, interventions, and outcomes. We included all adult patients up to age of 89, with an ICU length of stay of at least 24 h and having had at least one CAM-ICU assessment, as shown in the cohort selection diagram (Supplementary eFigure 1). Patients have been admitted for multiple reasons to these ICUs. Their admission diagnoses have not been a factor in the selection of our patients.

### Delirium assessment

Observation window refers to the period where patient data are collected, and the model is derived. Prediction window refers to the period from when the observation window ends up to the onset of the outcome, CAM-ICU positive in our case. We observed patients from 0 to 12 h, 0 to 24 h and 0 to 48 h. We predicted the incidence of Delirium for the next 12, 24, 48, 72, and 96 h. The diagnosis of delirium was made when at least one CAM-ICU value was positive.^15^ In instances with multiple CAM-ICU assessments, onset of delirium was determined from the time of the first positive CAM-ICU.

### Variable selection

The rationale for selection of independent variables was based on their ability to predict delirium in prior literature, availability in our databases, ease of extracting and monitoring in a real-time environment. We identified 21 categorical or numerical variables classified into demographic data, vital signs, laboratory values, and vasopressor dose that fulfilled above criteria.^24–35^ We also calculated daily sequential organ failure assessment (SOFA) scores to provide overall patient status. Since admission diagnoses or past medical history were not consistently available in the applied datasets, we excluded them. Downstream variables such as outcomes would not be available in real-time and similarly excluded. Initiation of delirium therapies like antipsychotic drugs could be a reaction to onset of delirium, and hence excluded to avoid confounding. Table 1 lists all the variables used.

**Table 1.** Variables included in the prediction models

Demographic data
Age, gender, height, weight
Vital signs
Oxygen saturation (SpO_2_), heart rate (HR), temperature
Other measurements
Sofa, sofa without GCS, Ventilation
Laboratory measurements
White blood cell count (WBC), sodium (Na), blood urea nitrogen (BUN), glucose, hemoglobin, platelets, potassium, chloride, bicarbonate, creatinine
Medications as continuous drips
Dopamine, epinephrine, norepinephrine, phenylephrine (all calculated as norepinephrine equivalent)

### Data preprocessing

All variables were aggregated into hourly intervals, where the last known value was used as a candidate for that interval. In cases where the last value for each variable is not measured in the interval, the representative of that interval was computed by averaging the available measurements in the interval. Missing values that were collected hourly like vital signs were imputed by forward and then if needed backward imputation. Categorical variables were converted into a vector to capture the semantics of each category at the model derivation phase. Specifically, we reshaped the data that was fed in 3 dimensions for BILSTM to 2-dimension input for Logistic Regression (LR) and Random Forest (RF) to have the same input for each model and to provide a fair comparison for each model. For all continuous variables, we utilized the recorded value in the database without any adaptation. Heat map further details the set of variables, including linear correlations between each variable (Supplementary eFigure 2).

### Model derivation and validation

We evaluated the results based on 5-fold stratified cross-validation. This method partitions the data into 5 equal segments. Respectively training and validation phases are done in 5 iterations in a manner that within each iteration a different segment of the data is held out for validation, while the remaining 4 segments are considered as a derivation set to train the model. Typically, metrics calculated based on the k-fold stratified cross-validation can effectively assess overfitting and has lower variance.^36^

We used 3 sets of algorithms to evaluate delirium prediction, namely LR, RF, and Bidirectional Long Short-Term Memory (BiLSTM). BiLSTM represents an evolution from recurrent neural network-based Long Short-Term Memory (LSTM), and with a backward input, preserves information from both past and future, producing more accurate predictions.^37^^,^^38^

Considering that both LR and RF are unable to process time series variables efficiently, we pre-processed the clinical variables and all-time steps and corresponding variables were flattened into a single record. This was done to ensure that both LR and RF have access to the same data about the changes in patient state as BiLSTM, to ensure a fair performance comparison.

### Statistical analysis

The classification results for delirium prediction are reported using the Area Under Receiver Operating Characteristic (AUROC), Area Under Precision Recall Curve (AUPRC), Recall, Precision (Positive Predictive Value), and Negative Predictive Value. Furthermore, we also investigated calibration quality of our models.

### Model interpretability

Although there are many definitions of interpretability, we focused on how the model ranks each input variable with respect to outcome prediction. LR and RF have been successfully employed in the clinical domain due to their ease of interpretation; however, they require additional processing to handle high-dimensional, longitudinal, and irregular electronic health record datasets.^39^ In this context, we employed the Shapley Value Sampling (SVS) method to probe the Bi-LSTM model.^40^ SVS is a perturbation-based method to compute variable attribution, which is based on sampling theory that can be used to estimate Shapley values.^41^ The SVS produces feature ranking with respect to each feature input, allowing us to rank these variables based on their predictive power. Given that interpretability of neural networks is still an open research question, especially for temporal neural networks, we also provide results from 2 other methods, namely Integrated Gradient and Guided Backpropagation, to ensure that the variable importance results are consistent across the 3 methods.^42–44^

### Source code

The entire code is available at https://github.com/mostafaalishahi/Delirium_prediction_models.

## RESULTS

### Patient characteristics

The eICU-CRD cohort consisted of 16 546 patients, with a mean age of 62.84 (±16.02) years and 46.53% were female. The incidence of delirium was 19.06% (Table 2). In the first 48 h of admission, 59.30% of patients presented with delirium. The MIMIC-III cohort consisted of 6294 patients, with a mean age of 63.58 (±15.79) years and 43.82% were female. The incidence of delirium was 20.15% and 66.34% of patients presented within the first 48 h of ICU admission (Table 2, Supplementary eFigure 3). For vital signs and laboratory values that were generated hourly, an average of 7% were missing values (Supplementary eTable 1). Patients with CAM-ICU+ status had a lower SOFA score, higher blood urea nitrogen, and white blood cell count.

**Table 2.** Characteristics of the included patients divided by their CAM-ICU status

Variable	eICU			MIMIC		
	CAM-ICU +	CAM-ICU −	*P* value	CAM-ICU +	CAM-ICU −	*P* value
Number of patients (%)	3153 (19)	13393 (81)	—	1268 (20)	5026 (80)	—
Age, mean (SD), years	65.53 (15.14)	62.20 (16.16)	<.05	64.81 (15.62)	63.27 (15.82)	<.05
Female (%)	1405 (44)	6295 (47)	—	545 (43)	2211 (44)	—
Height, mean (SD), m	168.47 (18.23)	169.25 (15.90)	<.05	170.06 (14.22)	168.88 (14.87)	.054
Weight, mean (SD), kg	83.06 (29.88)	85.00 (25.58)	<.05	82.68 (30.25)	81.53 (24.89)	0.15
Heart rate, mean (SD), bpm	88.22 (18.06)	85.09 (17.73)	<.05	88.60 (17.53)	85.12 (17.29)	<.05
Oxygen saturation, mean (SD), %	97.16 (2.72)	96.80 (2.79)	<.05	97.17 (2.71)	96.58 (4.50)	<.05
Glucose, mean (SD), mg/dL	140.32 (45.97)	146.46 (56.31)	<.05	144.51 (58.70)	141.25 (51.43)	<.05
Temperature, mean (SD), °C	37.01 (0.69)	36.97 (2.65)	<.05	37.06 (0.76)	36.88 (0.76)	<.05
Serum sodium, mean (SD), mEq/L	140.32 (5.80)	138.57 (5.04)	<.05	139.39 (5.48)	138.32 (4.89)	<.05
BUN, mean (SD), mg/dL	31.93 (22.10)	25.88 (18.64)	<.05	33.96 (24.46)	28.10 (20.77)	<.05
WBC, mean (SD), per microliter	13.01 (6.47)	11.08 (5.51)	<.05	12.13 (7.73)	10.74 (6.29)	<.05
Hemoglobin, mean (SD), g/dL	9.73 (1.89)	10.00 (2.08)	<.05	9.76 (1.68)	10.27 (1.76)	<.05
Platelets, mean (SD), per microliter	201.34 (122.76)	210.23 (108.70)	<.05	202.59 (137.23)	199.53 (114.33)	<.05
Serum potassium, mean (SD), mEq/L	3.98 (0.59)	4.00 (0.57)	.1431	4.03 (0.57)	4.07 (0.56)	<.05
Chloride, mean (SD), mEq/L	105.54 (6.86)	103.24 (6.29)	<.05	104.57 (6.69)	104.36 (6.37)	<.05
Serum bicarbonate, mean (SD), mEq/L	35.23 (5.02)	25.52 (5.02)	<.05	25.16 (5.21)	24.88 (4.95)	<.05
Serum creatinine, mean (SD), mg/dL	1.45 (1.16)	1.37 (1.21)	<.05	1.63 (1.28)	1.37 (1.05)	<.05
Ventilation, mean (SD)	0.87 (0.34)	0.71 (0.45)	<.05	0.56 (0.50)	0.33 (0.47)	<.05
Total norepinephrine dose (SD), mcg/kg/min	0.02 (0.31)	0.01 (0.28)	<.05	0.08 (0.63)	0.06 (0.57)	<.05
SOFA, mean (SD)	4.9 (3.3)	3.42 (2.84)	<.05	6.46 (3.77)	6.67 (3.34)	<.05
SOFA without GCS, mean (SD)	3.27 (2.83)	2.58 (2.33)	<.05	5.42 (3.65)	4.99 (3.13)	<.05

CAM-ICU: confusion assessment method in the ICU; +: present; −: absent; SD: standard deviation; m: meter; kg: kilogram; bpm: beats/minute; mg/dL: milligrams/deciliter; °C: degree Celsius; mEq/L: milli equivalents per liter; g/dL: gram per deciliter; mcg/kg/min: micrograms per kilogram per minute; SOFA: sequential organ failure assessment; BUN: Blood urea nitrogen; WBC: white blood cell count; GCS: Glasgow coma scale.

### Performance of machine learning models

**Figure 1.**
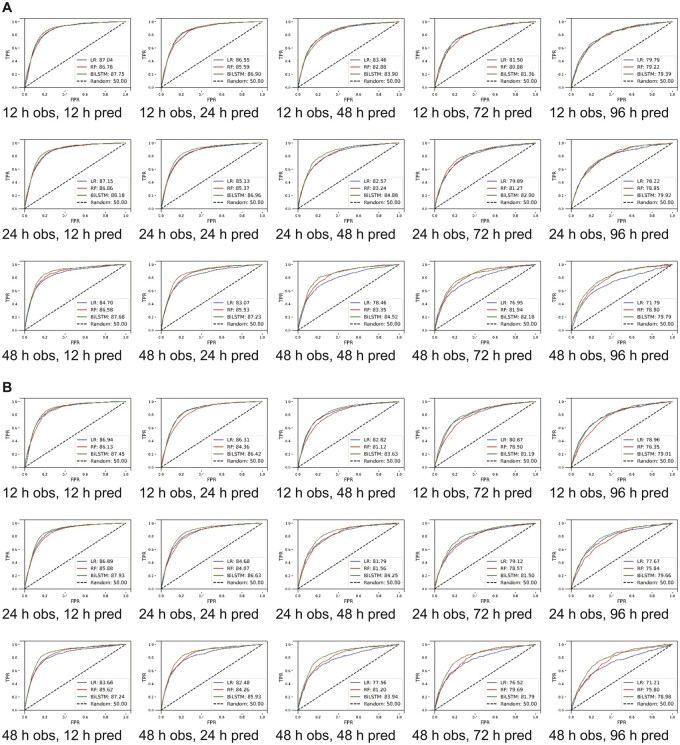
Model derived and validated using cross-validation. (A) Unmodified thresholds and (B) thresholds optimized for higher recall. AUROC: area under receiver operating curve; h: hour; obs: observation window; pred: prediction window; TPR: true positivity rate; FPR: false positivity rate; LR: logistic regression; RF: random forest; c: long short-term memory.

The BiLSTM algorithm was noted to have had the highest AUROC and AUPRC values for most of the observation-prediction combinations. The best performance on the eICU-CRD cohort was achieved with a 24 h observation and a 12-h prediction window, where AUROC of BiLSTM model was 88.39% (95% confidence interval [CI], 86.41–89.96) as shown in Supplementary eTable 2. Increasing the prediction window to 48 h (while keeping the observation window to 24 h), the AUROC of BiLSTM model was 84.87% (95% CI, 83.32%–86.41%), LR 82.57% (95% CI, 79.64%–85.47%), and RF 83.24% (95% CI, 81.83%–84.67%), and AUPRC of 34.97% (95% CI, 32.22%–37.27%), 31.07% (95% CI, 27.62%–33.81%), and 32.82% (95% CI, 28.89%–36.75%), respectively (Figure 1).

**Figure 2.**
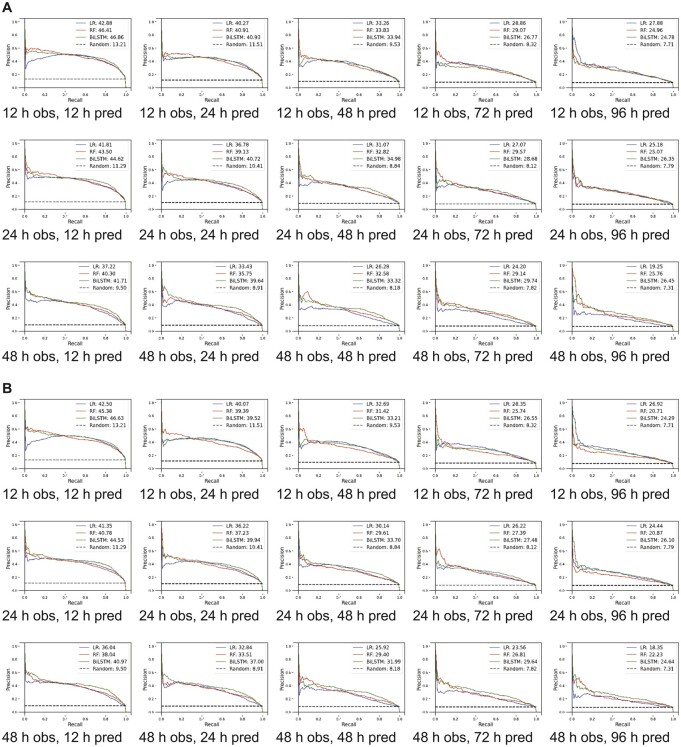
Model derived and validated using cross-validation. (A) Unmodified thresholds and (B) thresholds optimized for higher recall. AUPRC: area under precision recall curve; h: hour; obs: observation window; pred: prediction window; LR: logistic regression; RF: random forest; LSTM: long short-term memory.

Since BiLSTM had the best AUROCs and AUPRCs, we calculated the precision and recall values in each observation-prediction window using BiLSTM. In the eICU-CRD derivation cohort, for the 12-h observation window, the precision and recall decreased from 37.52% (95% CI, 36.00%–39.05%) to 28.68% (95% CI, 24.88%–32.49%) and from 86.1% (95% CI, 82.49%–89.71%) to 63.49% (95% CI, 52.91%–74.08%) respectively when the prediction window changed from 12 to 96 h (Figure 2 and Supplementary eTable 2). When increasing the observation window for 48-h prediction, the precision and recall changed from 32.82% (95% CI, 29.6%–36 .04%) to 17.9% (95% CI, 15.37%–20.44%) and from 82.22% (95% CI, 78.16%–86.27%) to 73.95% (95% CI, 64.8%–83.11%).

**Figure 3.**
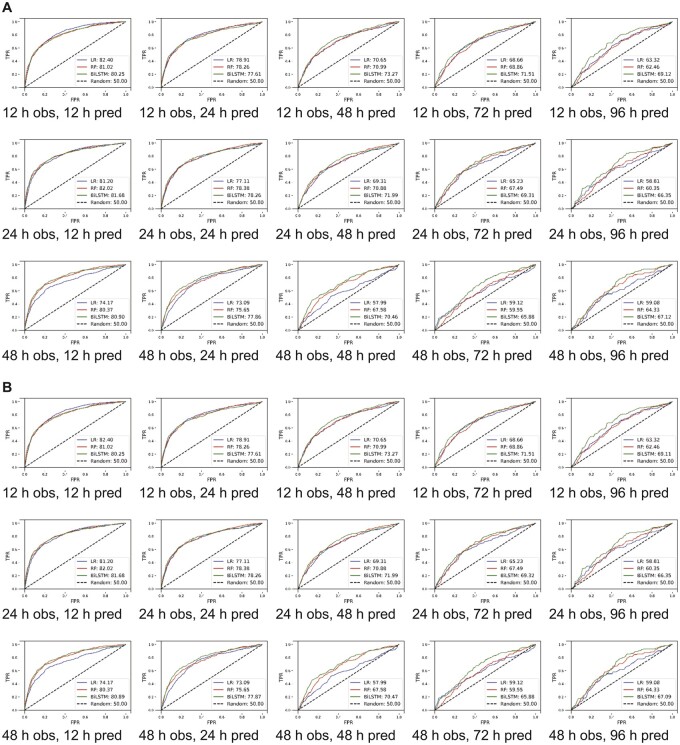
Model derived and validated using cross-validation. (A) Unmodified thresholds and (B) thresholds optimized for higher recall. AUROC: area under receiver operating curve; h: hour; obs: observation window; pred: prediction window; TPR: true positivity rate; FPR: false positivity rate; LR: logistic regression; RF: random forest; LSTM: long short-term memory.

**Figure 4.**
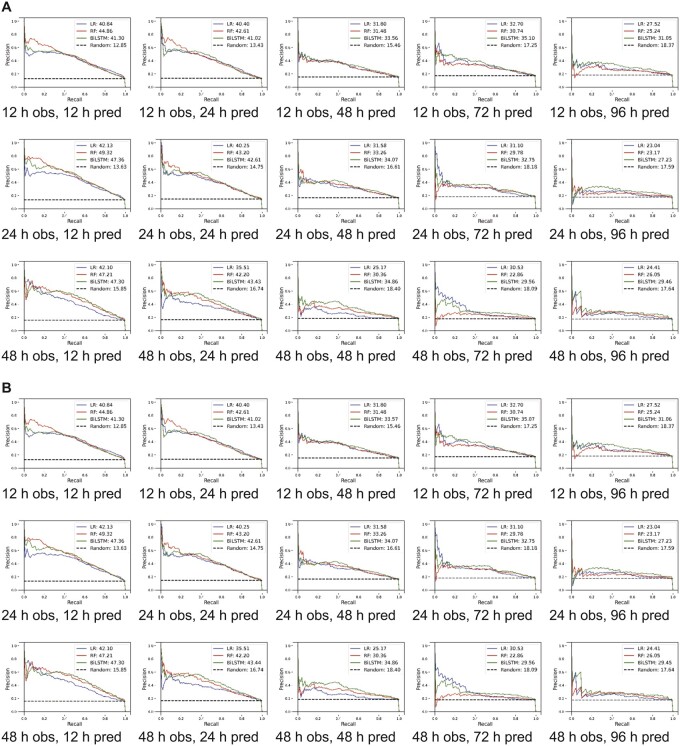
Model derived and validated using cross-validation. (A) Unmodified thresholds and (B) thresholds optimized for higher recall. AUPRC: area under precision recall curve; h: hour; obs: observation window; pred: prediction window; LR: logistic regression; RF: random forest; LSTM: long short-term memory.

As we were interested in making our model more sensitive for screening, we changed thresholds to have higher recall at the expense of precision, as such we assigned higher weights to the minority class (delirium positive).^45^ For a 12-h observation window, while recall changed slightly from 93.73% (95% CI, 93.1%–94.37%) to 92.57% (95% CI, 88.19%–96.95%) as the prediction window changed from 12 to 96 h, the precision decreased from 26.96% (95% CI, 24.99%–28.94%) to 11.34% (95% CI, 10.71%–11.98%) (Supplementary eTable 2). For the 48-h prediction window as we increased the observation window from 12 to 48 h, the precision and recall changed from 16.82% (95% CI, 15.61%–18.02%) to 15.64% (95% CI, 13.96%–17.42%) and 92.15% (95% CI, 88.47%–95.82) to 91.13% (95% CI, 89.57%–92.69%), respectively. Comparable results for the MIMIC-III cohort with varying thresholds are presented in Supplementary eTable 3, Figures 3 and 4. A heat map demonstrating correlation among features is presented in Supplementary eFigure 2 for the eICU-CRD for the MIMIC-III populations.

### Interpretability

**Figure 5.**
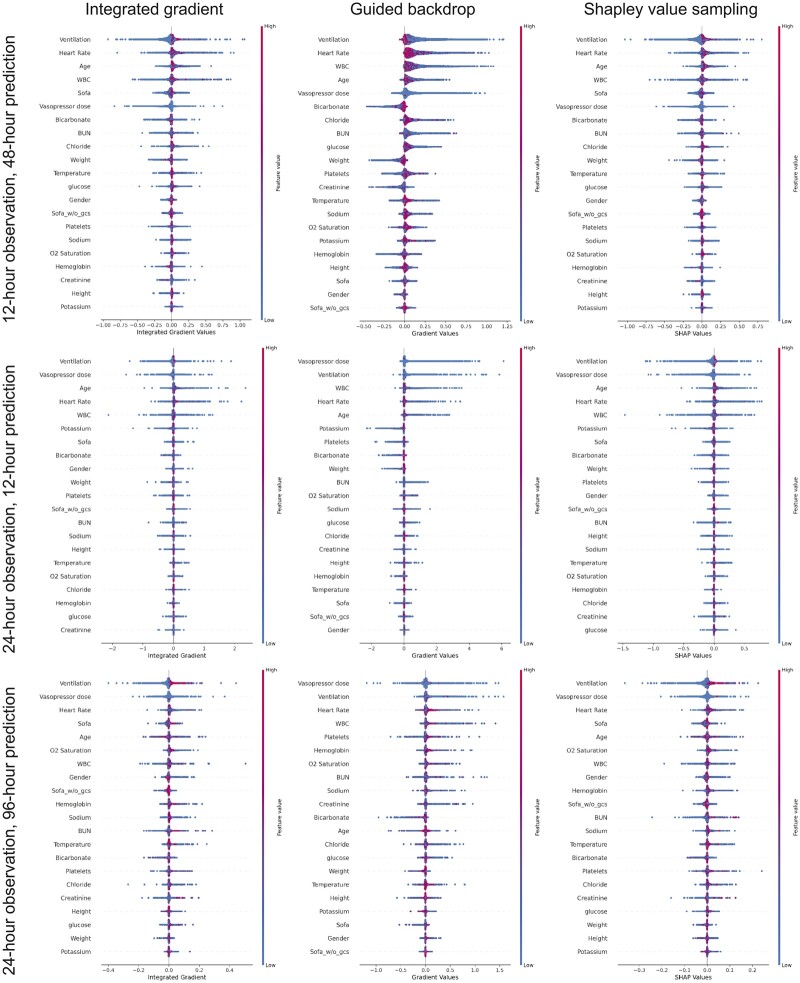
Features ranked according to their importance in descending order in long short-term memory model in eICU. Color shows whether ranked variable value is high (red) or low (blue) for that observation.

**Figure 6.**
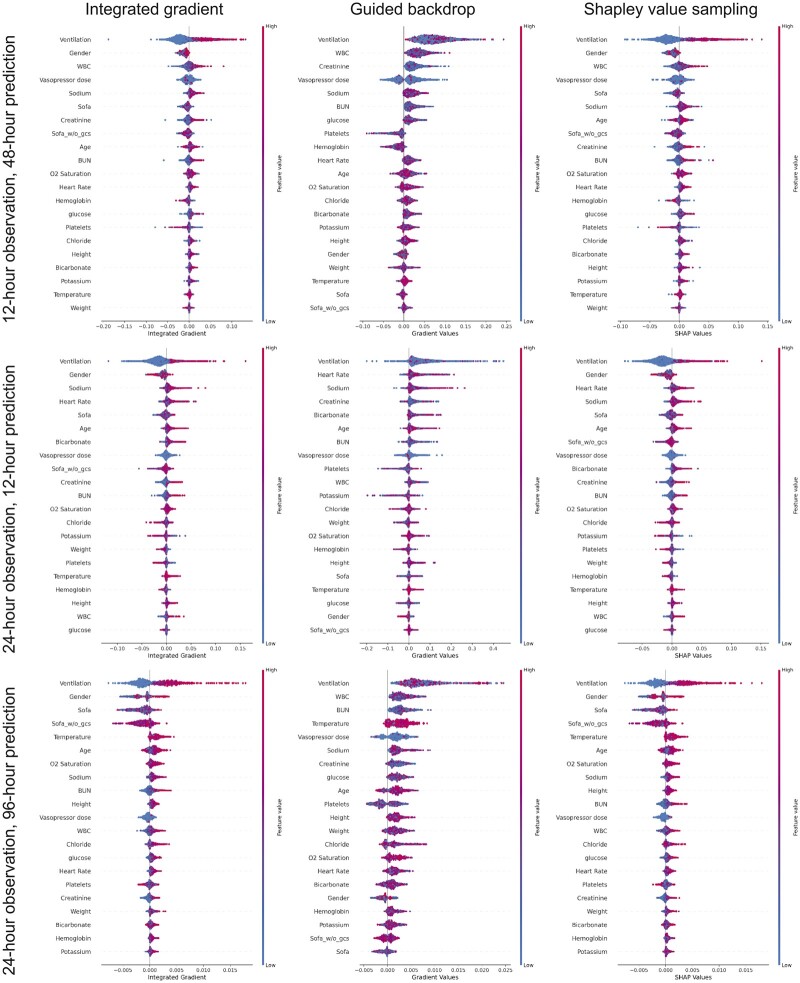
Features ranked according to their importance in descending order in long short-term memory model in MIMIC-III. Color shows whether ranked variable value is high (red) or low (blue) for that observation.

Figure 5 ranks the features that have contributed to delirium prediction according to their relative importance in the eICU-CRD derived model. Ventilation, heart rate, age, white blood cell count, SOFA score, and vasopressor use are the highest ranked features across different prediction windows. Most of these features are also the highest ranked features when assessing interpretability in the MIMIC III cohort (Figure 6).

## DISCUSSION

Our study shows that a machine learning model using only a few routine clinical variables replicated the performance of previously reported models that were developed using hundreds of variables. Our study successfully demonstrated that we could modify the performance of a model to fit our clinical needs as an effective screening tool. We took the following steps that helped us achieve our goal: (1) we studied the peak delirium onset time in our population and optimized the model to maximize predictive accuracy in that time frame; (2) we incorporated sliding windows in our model for continuous prediction across time and address drop in performance associated with predictions further ahead; and (3) we adjusted our thresholds to favor a high recall to ensure the model detects all patients at risk of delirium. Furthermore, we demonstrated that performance across different datasets diminish in accuracy and needs to be individualized to the population. Our features when ranked suggest older and more critically ill patients are at greater risk of delirium, especially in combination with mechanical ventilation and vasopressor therapy. Our model’s ranking of features is consistent with what we already know as high-risk features. We have shared our code for replicating the results and recommend adjustments be made according to the specific setting and their needs.^46^

Screening tools like CAM-ICU describe a snapshot in time and do not give an idea of the patient’s progress nor are predictive. Strategies based on established best practices such as ABCDEF are resource intensive and challenging to implement universally.^12^ Despite effective prevention strategies, delirium is still commonplace in the ICU highlighting a need for a screening tool that prioritizes patients at risk and allows us to exclude patients who are low risk from these time-consuming therapies. Few models exist that can both accurately predict and be easy to implement. Most models use several hundred variables or use only a snapshot of features that can vary with time. Also, many of these models were trained on small datasets and use inconsistent approaches for collecting and/or stratifying data into training and validation cohorts limiting generalizability.^19–21^ The Pre-Deliric and e-Pre-Deliric, were built with a handful of predictor variables from a large patient cohort, and been externally validated.^47^^,^^48^ However, they employ data from admission variables that change and lose predictive power with time. Recent machine learning-based algorithms were able to predict delirium accurately but using over 700 predictor variables and were also criticized for their analysis methods.^21^^,^^49^ Importantly, these models have not investigated how their performance changes with different prediction windows, optimal time of observation, capture the evolution of a patient’s state through time and unable to adjust delirium risk estimation temporally. In our knowledge, our report is one of the first instances of delirium prediction, where we have not only tried to predict accurately across different scenarios but also addressed the issues with prior prediction models. Notably, we have iteratively developed our model to address the challenges that are posed by low incidence of delirium, temporal progression of disease, and different patient populations. Additionally, we have ventured into the realm of explaining how our features contribute, something that is rare in models using deep learning.

The BiLSTM-based model, which has the advantage of capturing temporal dependencies, performed the best of the 3 models evaluated, suggesting that the trajectory of predictive features is more informative than a single value. The simpler LR model is an attractive option if implementation is determined by computational limitations of a deep learning model. A longer observation window gained little in terms of model performance. A 48-h observation window even led to a drop in accuracy, but this is due to a decrease in the size of the training cohort. Another possibility is that factors contributing to delirium are proximal to its onset, further justifying the use of continuous prediction using a sliding window. The decay in performance of the algorithm as it predicts delirium with longer lead time is similar in both MIMIC-III and eICU-CRD.

A screening tool needs to be sensitive. This is best addressed by a model with a high recall. We adjusted thresholds favoring a high recall while sacrificing precision (Supplementary eTables 2 and 3) to achieve this purpose. Also, it is desirable to have prediction algorithms that have short observation duration and predict the furthest ahead. In our case, since most (59% in eICU-CRD, 66% in MIMIC-III) delirium cases occurred within 48 h of ICU admission (Supplementary eFigure 3), hence we targeted performance for a 48-h prediction window with a 12- or 24-h observation window. We also demonstrated that as the prediction window moved beyond 48 h the model-maintained recall, but with a precipitous drop in precision. Non-trivial tuning of hyperparameters is required when algorithms are ported across populations. We suggest the performance of different observation and prediction times be studied on the local patient population and depending on the objective of the algorithm the optimal windows are determined. Furthermore, adapting the model to the local patient population could not only improve the predictive performance, but also calibration quality (Supplementary eFigure 4).

Delirium is precipitated through many factors, some that are unique to the ICU. Our variables were chosen *a priori* based on literature review. We only included variables that can be easily extracted in real time. Instead of using static values, we employed a sliding window for prediction and incorporated the trajectory of each variable over time. Our results indicate that this strategy predicts delirium more accurately than values captured at a moment in time and eliminates the need for long-term prediction.

Since we conducted a retrospective study, causality between the features and delirium cannot be established. Other limitations include selection bias (we excluded observations with missing CAM-ICU values) and interpreter bias (the data recorded in the databases might have been collected after the onset of delirium, given the discontinuous nature of CAM-ICU measurement). Additionally, CAM-ICU was scored by different nurses at separate times and in different units, potentially resulting in inter-operator variability. This study also does not have the ability to predict the duration or outcomes of each patient once delirium has occurred.

## Conclusion

We successfully designed a delirium prediction model as a potential screening tool for ABCDEF bundle implementation. Using a few clinically relevant predictor variables we were able to achieve comparable performance to contemporary and well-reported models. We were able to tackle the challenge presented by evolving temporal and treatment effects by using methods that captured temporal trends in data rather than static values and sliding observation windows, threshold adjustments to ensure consistently high recall. Additionally, we peeked at interpreting the model and shared our code online for reproducibility. We believe our model will help with identifying patients at risk of delirium early and will allow us to target preventive therapies, which is often time-consuming and personnel-intensive, to the patients who are most likely to benefit.

## FUNDING

This research received no specific grant from any funding agency in the public, commercial, or not-for-profit sectors.

## AUTHOR CONTRIBUTIONS

AB, AD, LAC, and VO participated in the study design. SS and WY performed the data analysis. All authors contributed in the analysis of results, manuscript preparation and have approved the final manuscript.

## SUPPLEMENTARY MATERIAL

Supplementary material is available at *JAMIA Open* online.

## CONFLICT OF INTERESTS STATEMENT

The authors have no competing interests to declare.

## DATA AVAILABILITY

The data underlying this article are available in eICU collaborative research database at https://dx.doi.org/doi:10.1038/sdata.2018.178 and MIMIC-III database at https://dx.doi.org/doi:10.1038/sdata.2016.35. The datasets were derived from sources in the public domain at https://physionet.org/content/eicu-crd/2.0/ and https://physionet.org/content/mimiciii-demo/1.4/, respectively.

## Supplementary Material

ooac048_Supplementary_Data
