# Supplementary material for: Delirium prediction in the ICU: designing a screening tool for preventive interventions
Source: JAMIA Open. 2022 Jun 10;5(2):ooac048. doi: 10.1093/jamiaopen/ooac048 (PMC9185728; doi:10.1093/jamiaopen/ooac048)
Supplement: ooac048_Supplementary_Data [file ooac048_supplementary_data.docx]

Contents:

eFigure1: Consort Diagram

eFigure2: Heat-map showing correlation between variables

eFigure3: Delirium incidence by day

eTable1: Frequency of Imputed values

eTable2: Performance metrics of derived model in eICU-CRD cohort.

eTable3: Performance metrics of derived model in MIMIC-III cohort.

eFigure4: Calibration curves for machine learning models

eTable4: Brier scores

eFigure 1: Consort Diagram


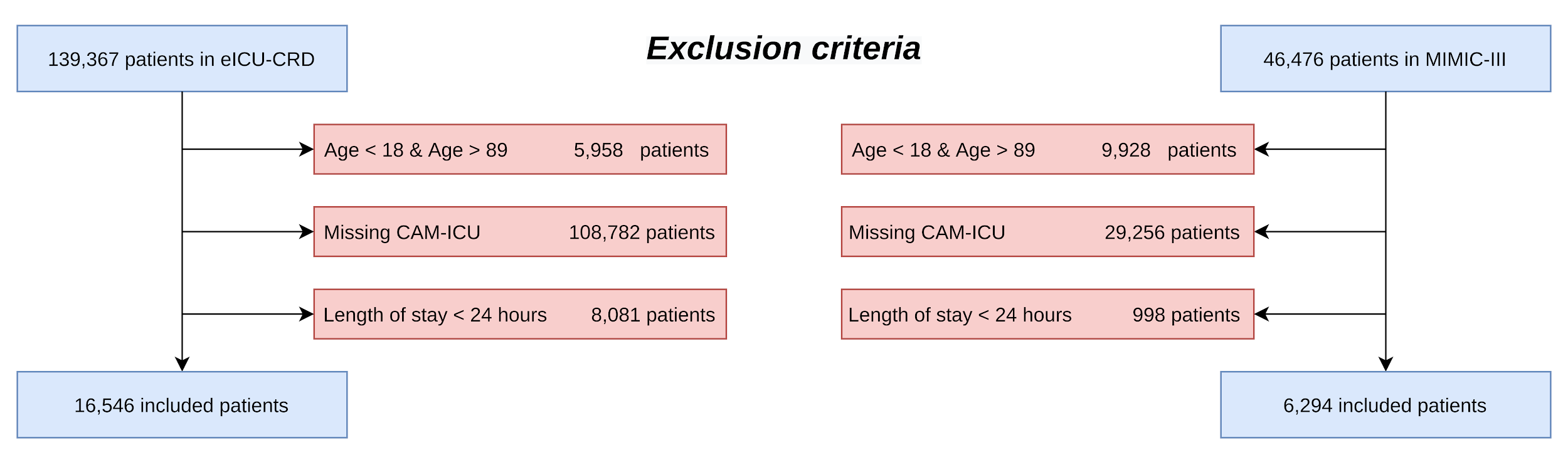


Left: eICU-CRD, Right: MIMIC III

eFigure 2: Heat-map showing correlation between variables.

| A. | B. |
| --- | --- |
| 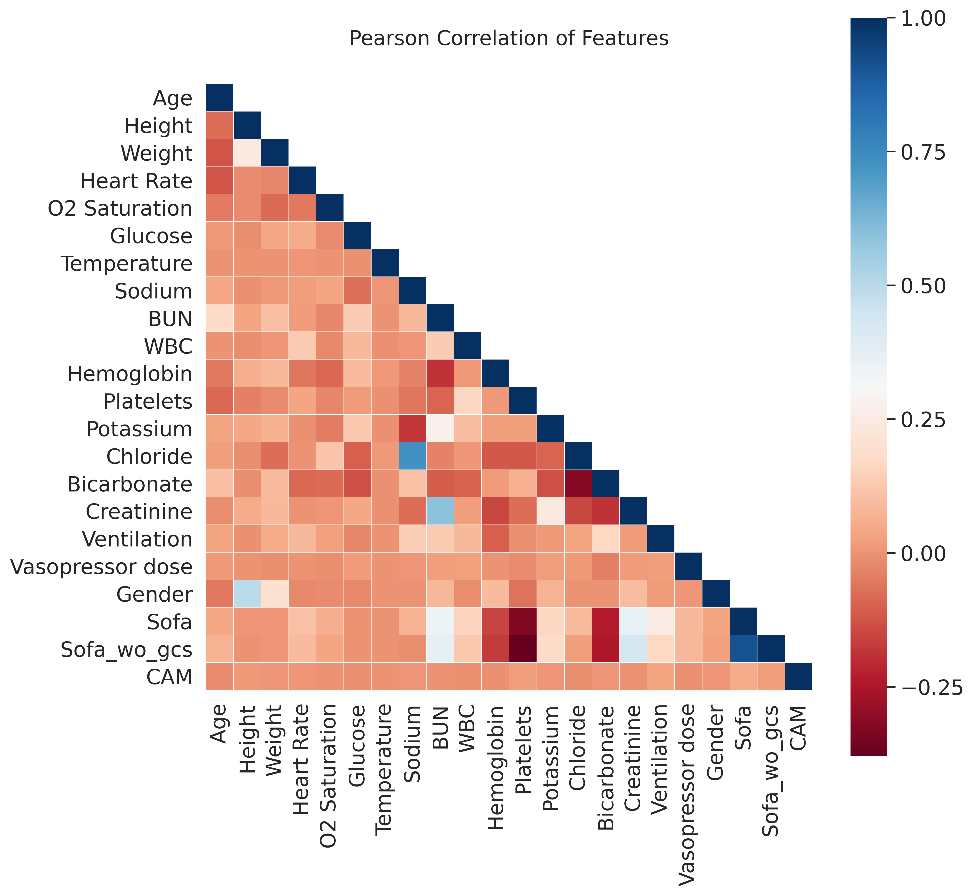 | 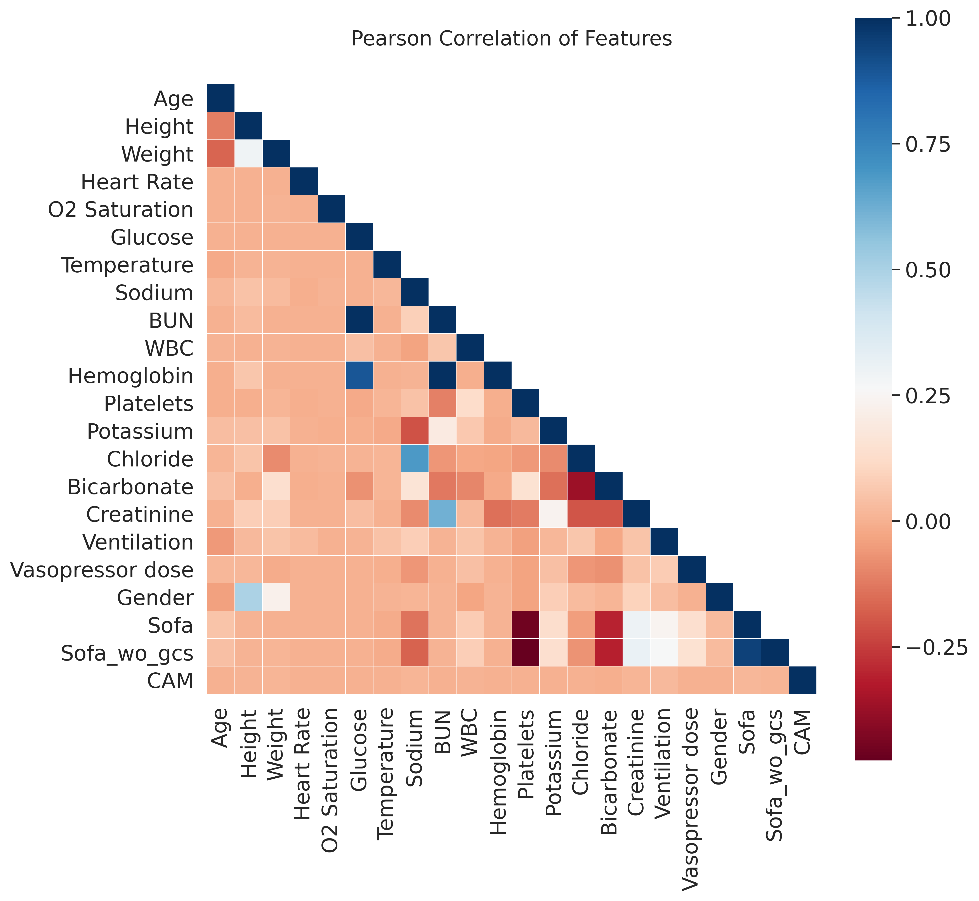 |

Blue shows strong positive correlation, Red shows strong negative correlation. Panel A: eICU-CRD, Panel B: MIMIC-III

eFigure 3: Delirium incidence by day

| A. | B. |
| --- | --- |
| 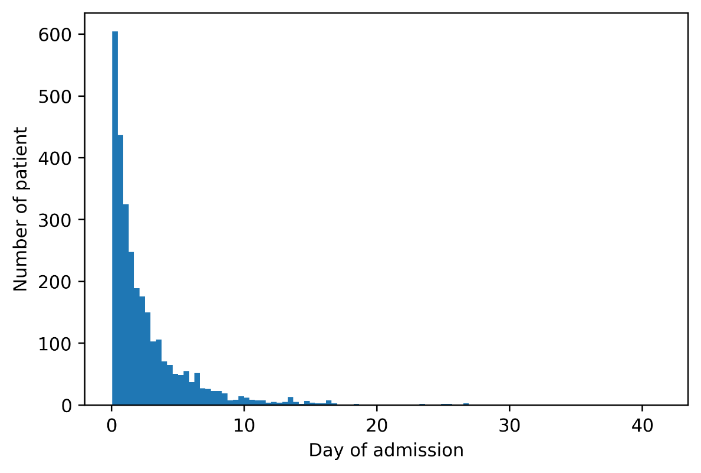 | 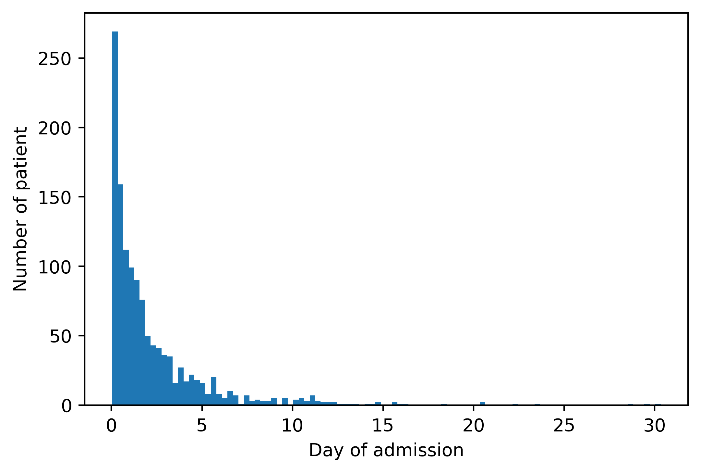 |

Panel A: eICU-CRD, Panel B: MIMIC-III

eTable 1: Frequency of Imputed values

| Variable | eICU (%) | MIMIC-III (%) |
| --- | --- | --- |
| Serum Bicarbonate | 2.18 | 14.58 |
| BUN | 0.54 | 9.30 |
| Chloride | 0.53 | 13.65 |
| Serum creatinine | 0.61 | 14.53 |
| Glucose | 0.34 | 5.71 |
| Heart Rate | 2.00 | 0.05 |
| Hemoglobin | 0.59 | 10.21 |
| Oxygen Saturation | 2.05 | 0.13 |
| Platelets | 1.65 | 16.04 |
| Serum Potassium | 0.51 | 13.49 |
| Total norepinephrine dose | 0.00 | 0.00 |
| Serum Sodium | 0.51 | 13.57 |
| SOFA | 0.00 | 0.00 |
| SOFA without GCS | 0.00 | 0.00 |
| Temperature | 0.09 | 4.08 |
| Ventilation | 0.00 | 0.00 |
| WBC | 0.65 | 15.52 |

Abbreviations: %: percentage, BUN: blood urea nitrogen, SOFA: sequential organ failure assessment, GCS: Glasgow coma scale, WBC: white blood cell.

eTable 2: Performance metrics of derived model in eICU-CRD cohort.

| Prediction window | 12 hours | 24 hours | 48 hours | 72 hours | 96 hours |
| --- | --- | --- | --- | --- | --- |
| Observation window |  |  |  |  |  |
| **A.** | **Unmodified Threshold** | | | | |
|  | Unmodified threshold: Area under receiver operating curve | | | | |
| 12 hours (95% CI), % | 87.82 (87.17-88.30) | 86.82 (85.15-88.64) | 84.00 (81.68-86.13) | 81.45 (78.61-84.10) | 79.03 (76.69-82.11) |
| 24 hours (95% CI), % | 88.39 (86.41-89.96) | 86.68 (85.79-88.15) | 84.87 (83.32-86.41) | 81.99 (80.66-83.38) | 79.93 (76.57-83.34) |
| 48 hours (95% CI), % | 88.00 (75.78-89.59) | 87.23 (86.30-88.20) | 84.51 (82.14-86.92) | 82.19 (80.99-83.41) | 79.78 (75.37-84.25) |
|  | Unmodified threshold: Area under precision recall curve | | | | |
| 12 hours (95% CI), % | 46.86 (42.52-50.85) | 40.92 (37.03-44.46) | 34.04 (28.99-38.24) | 26.78 (25.24-27.71) | 24.90 (18.48-30.22) |
| 24 hours (95% CI), % | 44.62 (39.11-50.02) | 40.85 (38.38-43.10) | 34.97 (32.22-37.27) | 28.68 (23.78-33.02) | 26.37 (21.00-31.28) |
| 48 hours (95% CI), % | 41.67 (37.52-45.62) | 39.64 (37.00-42.07) | 33.35 (27.58-38.88) | 29.75 (26.06-32.90) | 26.43 (19.65-32.71) |
|  | Unmodified threshold: Precision | | | | |
| 12 hours (95% CI), % | 37.52 (36.00-39.05) | 32.68 (29.09-36.28) | 25.01 (22.73-27.28) | 21.30 (20.09-22.49) | 17.45 (15.83-19.08) |
| 24 hours (95% CI), % | 35.27 (33.51-37.03) | 30.69 (28.71-32.66) | 24.84 (23.35-26.32) | 20.69 (18.24-23.15) | 19.08 (17.85-20.31) |
| 48 hours (95% CI), % | 32.82 (29.60-36.04) | 29.37 (25.18-33.56) | 24.17 (21.68-26.67) | 22.25 (67.85-82.37) | 17.90 (15.37-20.44) |
|  | Unmodified threshold: Recall | | | | |
| 12 hours (95% CI), % | 86.10 (82.49-89.71) | 84.09 (81.81-86.37) | 80.53 (76.76-84.30) | 77.96 (69.87-86.05) | 75.58 (68.33-82.83) |
| 24 hours (95% CI), % | 84.74 (81.57-87.90) | 83.87 (81.24-86.50) | 79.44 (75.53-83.35) | 78.73 (72.41-85.05) | 71.20 (61.95-80.45) |
| 48 hours (95% CI), % | 82.22 (78.16-86.27) | 82.06 (78.55-85.56) | 80.38 (75.53-85.24) | 75.11 (67.85-82.37) | 73.95 (64.80-83.11) |
| **B.** | **Threshold optimized favoring a higher recall** | | | | |
|  | Threshold optimized favoring higher recall: Area under receiver operating curve | | | | |
| 12 hours (95% CI), % | 87.45 (86.87-88.03) | 86.41 (84.12-88.71) | 83.63 (81.43-85.83) | 81.19 (78.49-83.89) | 79.01 (76.10-81.92) |
| 24 hours (95% CI), % | 87.93 (86.39-89.48) | 86.63 (85.41-87.86) | 84.25 (82.92-85.62) | 81.50 (80.13-82.90) | 79.66 (76.61-82.72) |
| 48 hours (95% CI), % | 87.24 (85.34-89.15) | 85.93 (84.29-87.60) | 83.94 (81.72-85.90) | 81.76 (81.03-82.61) | 78.99 (74.90-83.01) |
|  | Threshold optimized favoring higher recall: Area under precision recall curve | | | | |
| 12 hours (95% CI), % | 46.63 (42.17-50.93) | 39.52 (34.88-43.89) | 33.21 (28.84-36.83) | 26.55 (23.55-28.97) | 24.30 (18.52-29.21) |
| 24 hours (95% CI), % | 44.55 (39.40-49.02) | 39.95 (38.25-41.47) | 33.70 (30.96-36.07) | 27.49 (22.86-31.46) | 26.11 (22.02-29.85) |
| 48 hours (95% CI), % | 40.96 (36.55-44.72) | 36.98 (32.38-41.33) | 32.12 (26.50-37.45) | 29.55 (25.26-33.35) | 24.65 (15.60-33.20) |
|  | Threshold optimized favoring higher recall: Precision | | | | |
| 12 hours (95% CI), % | 26.96 (24.99-28.94) | 22.04 (20.66-23.42) | 16.82 (15.61-18.02) | 13.33 (13.03-13.60) | 11.34 (10.71-11.98) |
| 24 hours (95% CI), % | 23.61 (22.55-24.66) | 21.73 (20.63-22.83) | 16.57 (15.74-17.38) | 13.46 (12.29-14.62) | 12.60 (11.81-13.39) |
| 48 hours (95% CI), % | 23.18 (20.49-25.87) | 18.70 (14.49-22.87) | 15.64 (13.96-17.42) | 14.02 (12.06-16.04) | 11.69 (10.75-12.73) |
|  | Threshold optimized favoring higher recall: Recall | | | | |
| 12 hours (95% CI), % | 93.73 (93.10-94.37) | 93.08 (90.42-95.75) | 92.15 (88.47-95.82) | 92.08 (90.25-93.91) | 92.57 (88.19-96.95) |
| 24 hours (95% CI), % | 93.59 (91.69-95.48) | 92.29 (88.83-95.76) | 91.65 (89.07-94.23) | 89.72 (86.74-92.69) | 90.40 (88.58-92.23) |
| 48 hours (95% CI), % | 90.49 (86.48-94.50) | 91.46 (89.97-92.95) | 91.13 (89.57-92.69) | 89.37 (84.87-93.41) | 90.20 (82.79-97.61) |

Panel A. Unmodified thresholds. Panel B. After thresholds were optimized favoring higher recall. Abbreviations: eICU-CRD: eICU Collaborative Research Database, 95% CI: 95 percent confidence interval, %: percentage.

eTable 3: Performance metrics of LSTM model in MIMIC-III cohort.

| Prediction window | 12 hours | 24 hours | 48 hours | 72 hours | 96 hours |
| --- | --- | --- | --- | --- | --- |
| Observation window |  |  |  |  |  |
| **A.** | **Unmodified Threshold** | | | | |
|  | Area under receiver operating curve | | | | |
| 12 hours (95% CI), % | 80.34 (78.31-82.21) | 77.64 (75.92-79.28) | 73.38 (69.43-77.15) | 71.47 (66.24-76.77) | 69.21 (63.95-74.41) |
| 24 hours (95% CI), % | 81.72 (78.09-85.36) | 78.25 (75.97-80.63) | 72.14 (64.37-79.61) | 69.06 (61.33-77.29) | 66.26 (56.31-76.38) |
| 48 hours (95% CI), % | 81.15 (79.46-82.30) | 77.90 (74.96-80.84) | 70.38 (64.35-76.59) | 65.87 (58.36-73.44) | 67.20 (61.93-72.46) |
|  | Area under precision recall curve | | | | |
| 12 hours (95% CI), % | 41.61 (36.14-46.56) | 40.97 (34.96-46.05) | 33.52 (30.06-37.08) | 34.93 (29.39-39.89) | 31.16 (26.65-35.69) |
| 24 hours (95% CI), % | 48.00 (43.11-52.94) | 42.54 (36.27-48.58) | 34.19 (27.39-40.66) | 32.76 (24.17-41.03) | 27.29 (19.35-34.66) |
| 48 hours (95% CI), % | 48.08 (42.59-53.32) | 43.48 (36.68-50.22) | 34.15 (29.67-38.03) | 28.02 (22.75-32.56) | 29.33 (24.80-33.66) |
|  | Precision | | | | |
| 12 hours (95% CI), % | 30.14 (26.54-33.74) | 35.12 (31.85-38.39) | 30.99 (27.91-34.07) | 30.86 (26.90-34.82) | 28.68 (24.88-32.49) |
| 24 hours (95% CI), % | 34.07 (31.36-36.79) | 33.35 (29.82-36.88) | 30.21 (27.00-33.41) | 28.36 (23.08-33.65) | 24.71 (19.89-29.52) |
| 48 hours (95% CI), % | 36.05 (32.37-39.74) | 34.27 (32.22-36.32) | 30.61 (28.27-32.95) | 26.69 (21.06-32.32) | 26.92 (22.57-31.26) |
|  | Recall | | | | |
| 12 hours (95% CI), % | 71.75 (68.75-74.74) | 64.80 (57.11-72.49) | 65.36 (62.42-68.29) | 62.91 (58.26-67.57) | 63.49 (52.91-74.08) |
| 24 hours (95% CI), % | 73.93 (67.53-80.32) | 69.23 (66.58-71.89) | 65.38 (59.77-70.99) | 60.35 (49.13-71.57) | 60.42 (46.04-74.80) |
| 48 hours (95% CI), % | 74.35 (67.09-81.61) | 70.00 (66.75-73.25) | 64.04 (53.35-74.74) | 60.69 (48.72-72.66) | 64.00 (49.96-78.04) |
| **B.** | **Threshold optimized favoring a higher recall** | | | | |
|  | Area under receiver operating curve | | | | |
| 12 hours (95% CI), % | 80.25 (78.31-82.21) | 77.61 (75.92-79.28) | 73.27 (69.43-77.15) | 71.51 (66.25-76.76) | 69.12 (63.96-74.38) |
| 24 hours (95% CI), % | 81.67 (78.09-85.36) | 78.26 (75.97-80.63) | 71.99 (64.37-79.62) | 69.31 (61.33-77.28) | 66.35 (56.31-76.38) |
| 48 hours (95% CI), % | 80.89 (79.46-82.30) | 77.87 (74.96-80.83) | 70.47 (64.35-76.60) | 65.86 (58.35-73.44) | 67.09 (61.94-72.40) |
|  | Area under precision recall curve | | | | |
| 12 hours (95% CI), % | 41.30 (36.12-46.04) | 41.02 (35.64-45.60) | 33.56 (29.61-37.58) | 35.07 (29.90-39.51) | 31.07 (26.63-35.49) |
| 24 hours (95% CI), % | 47.35 (43.17-51.63) | 42.61 (36.53-48.48) | 34.07 (26.91-40.81) | 32.74 (24.69-40.53) | 27.24 (19.51-34.35) |
| 48 hours (95% CI), % | 47.30 (43.15-51.42) | 43.50 (36.80-50.05) | 34.86 (30.14-39.06) | 29.53 (21.64-36.90) | 29.44 (24.71-33.94) |
|  | Precision | | | | |
| 12 hours (95% CI), % | 20.98 (19.31-22.64) | 23.78 (20.54-27.01) | 21.67 (19.08-24.26) | 23.27 (21.34-25.21) | 23.30 (21.45-25.14) |
| 24 hours (95% CI), % | 25.67 (24.48-26.86) | 25.41 (21.16-29.65) | 23.09 (21.71-24.47) | 23.35 (21.12-25.57) | 20.90 (18.66-23.15) |
| 48 hours (95% CI), % | 28.08 (24.45-31.75) | 26.67 (25.20-28.14) | 24.57 (23.20-25.93) | 22.51 (19.89-25.05) | 23.70 (22.20-25.20) |
|  | Recall | | | | |
| 12 hours (95% CI), % | 86.63 (83.32-90.01) | 76.95 (73.08-80.82) | 81.46 (71.94-90.98) | 84.47 (77.72-91.22) | 87.38 (73.19-99.05) |
| 24 hours (95% CI), % | 82.22 (76.40-88.05) | 81.14 (79.88-82.40) | 87.36 (74.76-92.75) | 84.11 (74.56-93.66) | 86.14 (73.04-99.24) |
| 48 hours (95% CI), % | 83.18 (76.24-90.13) | 83.79 (76.92-90.66) | 82.24 (71.18-93.30) | 83.20 (70.13-96.27) | 87.38 (78.40-96.36) |

Panel A. Unmodified thresholds. Panel B. After thresholds were optimized favoring higher recall. Abbreviations: LSTM: long short term memory, SD: standard deviation, %: percentage.

eFigure 4: Calibration Graphs for Machine Learning Models

| **A.** | | | | |
| --- | --- | --- | --- | --- |
| 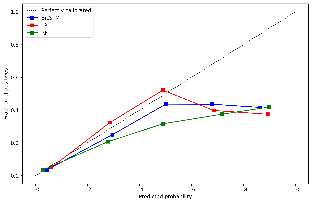 | 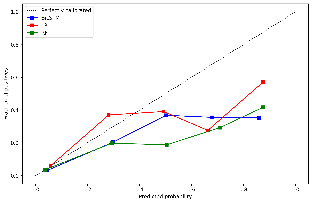 | 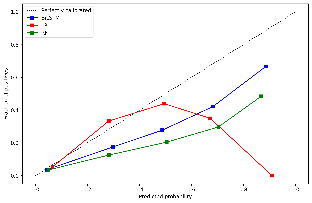 | 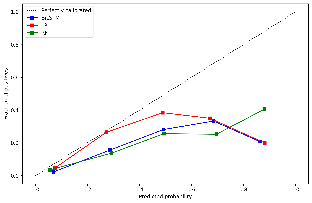 | 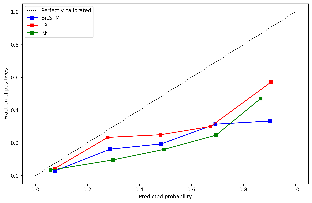 |
| 12 h obs, 12 h pred | 12 h obs, 24 h pred | 12 h obs, 48 h pred | 12 h obs, 72 h pred | 12 h obs, 96 h pred |
|  |  |  |  |  |
| 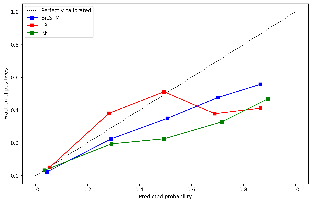 | 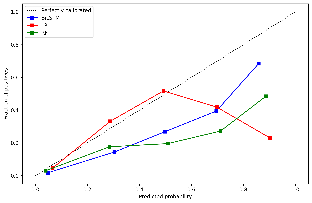 | 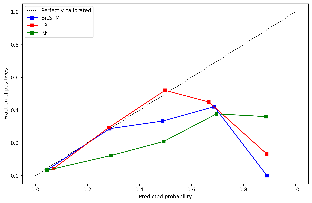 | 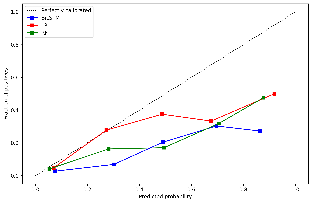 | 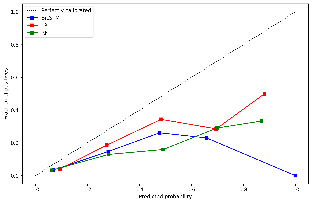 |
| 24 h obs, 12 h pred | 24 h obs, 24 h pred | 24 h obs, 48 h pred | 24 h obs, 72 h pred | 24 h obs, 96 h pred |
|  |  |  |  |  |
| 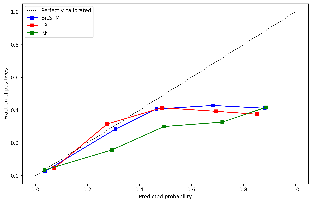 | 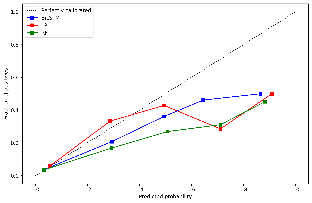 | 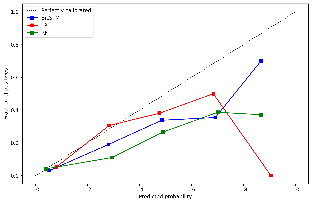 | 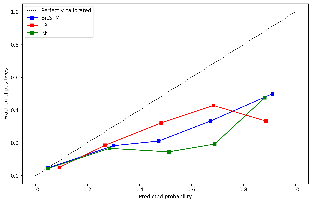 | 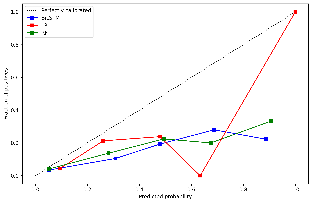 |
| 48 h obs, 12 h pred | 48 h obs, 24 h pred | 48 h obs, 48 h pred | 48 h obs, 72 h pred | 48 h obs, 96 h pred |
|  |  |  |  |  |
| **B.** | | | | |
| 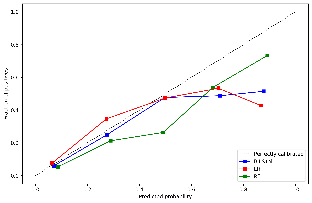 | 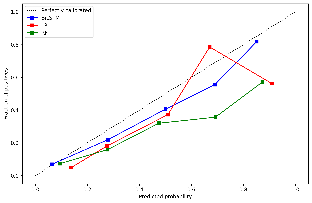 | 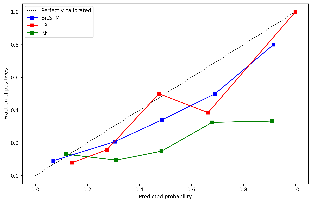 | 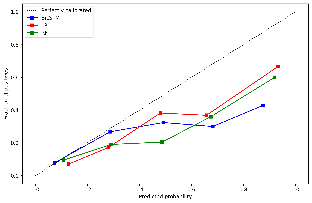 | 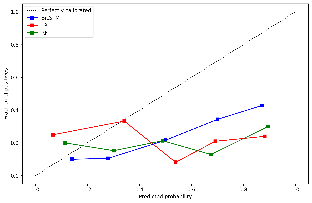 |
| 12 h obs, 12 h pred | 12 h obs, 24 h pred | 12 h obs, 48 h pred | 12 h obs, 72 h pred | 12 h obs, 96 h pred |
|  |  |  |  |  |
| 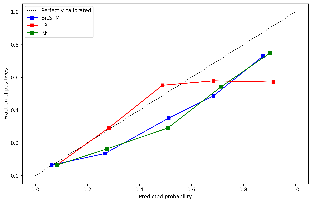 | 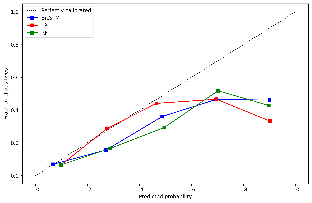 | 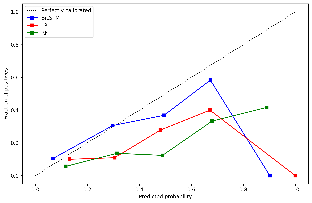 | 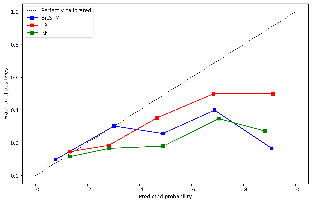 | 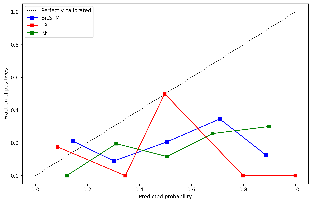 |
| 24 h obs, 12 h pred | 24 h obs, 24 h pred | 24 h obs, 48 h pred | 24 h obs, 72 h pred | 24 h obs, 96 h pred |
|  |  |  |  |  |
| 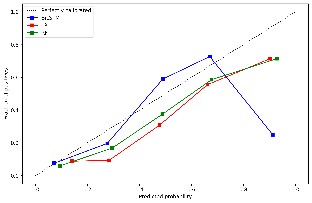 | 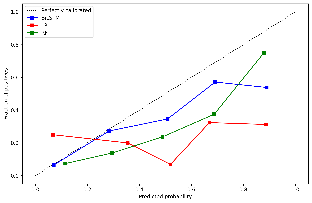 | 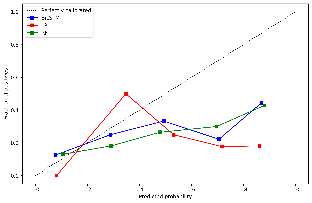 | 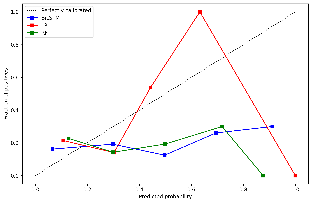 | 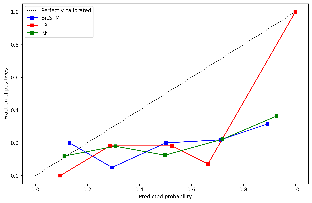 |
| 48 h obs, 12 h pred | 48 h obs, 24 h pred | 48 h obs, 48 h pred | 48 h obs, 72 h pred | 48 h obs, 96 h pred |
|  |  |  |  |  |

Panel A: eICU-CRD, Panel B: MIMIC III. Abbreviations: LR: logistic regression, RF: random forest, LSTM: long short term memory.

eTable 4: Brier Scores

| Observation Window | Prediction Window | BiLSTM | RF | LR |
| --- | --- | --- | --- | --- |
| 12 hours | 12 hours | 0.0912 | 0.0938 | 0.0945 |
| 12 hours | 24 hours | 0.0901 | 0.1058 | 0.0986 |
| 12 hours | 48 hours | 0.1161 | 0.1272 | 0.1179 |
| 12 hours | 72 hours | 0.1344 | 0.1365 | 0.1296 |
| 12 hours | 96 hours | 0.1390 | 0.1492 | 0.1483 |
| 24 hours | 12 hours | 0.0947 | 0.0961 | 0.0957 |
| 24 hours | 24 hours | 0.1075 | 0.1113 | 0.1125 |
| 24 hours | 48 hours | 0.1287 | 0.1322 | 0.1320 |
| 24 hours | 72 hours | 0.1442 | 0.1473 | 0.1447 |
| 24 hours | 96 hours | 0.1440 | 0.1459 | 0.1468 |
| 48 hours | 12 hours | 0.1109 | 0.1101 | 0.1206 |
| 48 hours | 24 hours | 0.1141 | 0.1256 | 0.1358 |
| 48 hours | 48 hours | 0.1481 | 0.1470 | 0.1511 |
| 48 hours | 72 hours | 0.1533 | 0.1509 | 0.1497 |
| 48 hours | 96 hours | 0.1428 | 0.1401 | 0.1423 |

Abbreviations: LR: logistic regression, RF: random forest, LSTM: long short term memory.
